# HRI-confusion: A multimodal dataset for modelling and detecting user confusion in situated human-robot interaction

**DOI:** 10.1016/j.dib.2025.112047

**Published:** 2025-09-10

**Authors:** Na Li, Jane Courtney, Robert Ross

**Affiliations:** a School of Computer Science, Technological University Dublin, Dublin, Ireland; b School of Electrical & Electronic Engineering, Technological University Dublin, Dublin, Ireland

**Keywords:** Human-robot interaction, Confusion, User social behaviours, Multimodal data

## Abstract

The dataset was collected from 28 participants (17 female, 9 male, and 1 non-binary) for a study aimed at modelling and detecting user social behaviours with different confusion states in task-oriented situated human-robot interaction (HRI). The dataset consists of user facial body video recordings synchronised with user speech across three designed experiment scenarios (Tasks 1 - 3). Each experiment lasted approximately one hour per participant. The videos are segmented into individual clips corresponding to specific experimental conversations under predefined conditions: general confusion and non-confusion for Task 1 and 3; and productive confusion, unproductive confusion, and non-confusion for Task 2.

In total, the dataset contains 789 video clips (body: 392, face: 397). Each video is recorded in high-definition RGB format, capturing user facial expressions or body language along with their speech. These multimodal data provide a valuable resource for studying user cognitive and mental states in human-robot interaction and human-computer interaction.

The data collected for Task 2 was used in [9]. In compliance with GDPR (General Data Protection Regulation) and DPIA (data protection impact assessment) guidelines, the dataset is freely available upon request at https://sites.google.com/view/hridatarequst/home.

Specifications Table

**Table:** 

Subject	Human-Computer Interaction, Human-Robot Interaction, Social and Personality Psychology, Robotics and Artificial Intelligence (AI), Linguistics
Specific subject area	Videos of 28 users engaging in oral interaction with a real social robot capturing their social behaviours, including facial expressions, body language and speech. Each video focuses on either facial expression or body language.
Type of data	Video (MP4)
Data collection	Two high-quality webcams were connected to OBS (Open Broadcaster Software) Studio software on an experimental laptop via two USB 3.0 ports for recording. One webcam was positioned behind the robot, facing the participant’s face, while the other was placed beside the robot to capture body language. Each participant wore a wearable microphone, which was also connected to the OBS studio software via the microphone jack on the same laptop to record user speech.
Data source location	Technological University Dublin, Grangegorman Campus, Dublin, Republic of Ireland. Latitude and longitude (and GPS coordinates) for collected data: (53.35656696401305, −6.281810843827172)
Data accessibility	Repository name: User Confusion-HRI Dataset Direct URL to request data: https://sites.google.com/view/hridatarequst/home Instructions for accessing these data: To access these data, the applicants must complete a request form using the link provided above. After the evaluation of their request, a temporary download link will be sent to the applicant via email.
Related research article	Li N and Ross R, Invoking and identifying task-oriented interlocutor confusion in human-robot interaction. Front. Robot. AI 10:1244,381 (2023). https://doi.org/10.3389/frobt.2023.1244381

## 1 Value of the Data

• The raw data presented in this work offers substantial value to social agent designers, enabling the development of AI agents that can interpret human mental states, particularly user confusion, in task-oriented interaction.
• Beyond being specifically used to study the manifestation of confusion states, social scientists, psychologists, and engineers may use these data to investigate other affective states, such as uncertainty, engagement, and emotional responses.
• These raw data include a total of 28h of user-robot interaction videos from 28 users, which can be used to test and benchmark relevant tasks of human-agent interaction systems, including emotion estimation, confusion classification, and cognitive load assessment.
• All facial video, body video, and audio recordings are temporally and spatially aligned, enabling comprehensive multimodal analysis.

## 2 Background

The study resulting in this dataset aimed to investigate and develop long-lasting and high-quality user engagement during interactions in HRI. User confusion, as a mental state, has been studied across various fields*, i.e*., mental health, psychology, learning [10]. Within the predominant studies on confusion in HCI, [4] specifically investigated confusion within online learning system. Theoretically, they proposed that confusion is a central state in complex learning processes, for example, modelling complex information and solving challenging problems. [10] proposed two distinct confusion states to capture different levels of user confusion: productive confusion and unproductive confusion in learning. They explained that productive confusion occurs when learners actively engage in solving their problems to overcome confusion, whereas unproductive confusion arises when learners are unable to resolve their problems, leading to persistent confusion.

Regarding our study of user confusion in HRI, we define confusion in HRI as “*a mental state in which, under certain circumstances, a human experiences obstacles in the flow of interaction. A series of behaviour responses (which may be nonverbal, verbal, and, or nonlinguistic vocal expression) may be triggered, and the human who is confused will typically want to solve the state of cognitive disequilibrium in a reasonable duration. If the confusion state is maintained for longer periods, the interlocutor may become frustrated or even drop out of the ongoing interaction.*” [7]. It should be noted that cognitive load as a factor impacts user's confusion states but that these are very distinct concepts. In a learning process, cognitive load can measure cognitive effort that is imposed by a task requirement [14,15]. Confusion also can be regarded as a type of uncertainty within educational settings [16]. Specifically, this uncertainty aligns with the concept of ``metaignorance state'', which describes a condition in which individuals recognise their own lack of knowledge [17]. Putting it simple, we can have a high cognitive load without being confused, and we can be confused even under a low cognitive load.

In the contexts of embodied interaction and specifically HRI and HCI, there are several existing datasets focused on user engagement and personality assessment. DAiSEE [6] is a multi-label video classification dataset designed to recognise learners’ affective states, including boredom, confusion, engagement, and frustration, in online learning environments. Turning to robot centric interaction, the UE-HRI [1] dataset captures spontaneous interactions between humans and a Pepper robot in public, providing rich data for various research areas. For example, the researchers developed deep learning techniques to detect real-time decreases in user engagement during HRI [2]. The MHHRI dataset [3] meanwhile enables comparative studies of personality and engagement in both human-human and human-robot interactions. It demonstrates that multimodal approaches generally produce better performance in classifying personality traits. Comparing large language models (LLMs) that can answer or assist users with dynamic text-based tasks, the HoloAssist dataset [13] is a larger-scale human interaction dataset comprising 166 h of recordings with 222 participants. This dataset contains natural human behaviours executed during 20 real-world procedural and physical manipulation tasks, where two people collaborated to complete the tasks. [12] studied the detection of user confusion on the HoloAssist dataset without user facial expressions during physical tasks when users face difficulty and require assistance.

While HoloAssist and other datasets do explore human-system interaction in various ways, the level of detail and amount of control in existing datasets is limited, highlights a need for focused, well designed datasets with clear research goals under controlled conditions. Our study specifically focuses on understanding user confusion behaviours in spoken, task-oriented interactions between humans and social robots, using a set of designed tasks to elicit confusion states. These tasks employed varying levels of verbal problems to induce different levels of user confusion in a controlled laboratory environment. In this regard, our dataset differs from existing resources. For example, in the UE-HRI dataset, user data were collected in public settings, and conversations were more spontaneous, with the primary focus on studying user engagement. Similarly, in the MHHRI dataset, the authors recorded two types of interactions—human–human and human–robot—designed in a manner similar to UE-HRI, with the aim of studying personality and engagement rather than confusion. In contrast, the HoloAssist dataset was used to study user confusion by detecting body language alone, without including facial data or speech. Furthermore, the tasks in HoloAssist did not involve social spoken interaction, whereas our dataset incorporates this element.

This dataset contains data from 28 users and is, therefore, a valuable resource for further research focused on testing hypotheses, descriptive analysis, or conversational analysis. Comparably sized and scoped datasets include Day et al. [5], who examined how users interact with small service robots for indoor human trajectory prediction. Their dataset, collected from 23 participants, includes two different agent types (robot and human) across seven scenarios, with the robot switching between three motion controllers under various permutations of human agents. Similarly, Zehfroosh et al. [20] employed a Markov decision process (MDP) to encode human intention as hidden states, treating human actions as observables. While datasets of this size are not used to train foundational models, focused multimodal interaction datasets can be used in model prototyping and evaluation activities.

Building on the success of our previous work [8], the dataset presented here provides a valuable resource for those wishing to study confusion and its manifestation across HCI and HRI. Regarding confusion, our work has followed the definition of [7], who explained that confusion in HCI can be interpreted as a unique state for evaluating user engagement by observing social behaviours. To provide clarity on the topic, and particularly in the context of HRI, our work presented a concrete study and data collection in the task-oriented HRI context for confusion elicitation and detection. In this earlier work, we also provided definitions for productive confusion and unproductive confusion in situated HRI [9].

Regarding experiment design (described in detail later), three tasks were performed by each participant, with a total duration of approximately one hour: two oral question-answer conversation (Tasks 1 and 2) and one “pick and place” game (Task 3). The multimodal dataset captured user facial expressions, body language and speech, using multiple devices to collect separate data streams. These data are categorised according to the designed experimental conditions: confusion/non-confusion conditions for Task 1 and 3, productive confusion/unproductive confusion/non-confusion conditions for Task 2.

To study user social behaviours in different confusion and non-confusion states, we successfully applied multiple measurement and analysis techniques on the data from Task 2 to extract multimodal feature data (*i.e.,* eye-gaze, head pose, facial emotion indexes, speech emotions, silence duration time and speech pitch values) and detect correlations with the experimental conditions [9]. In addition to providing a rich dataset, the dataset presented here provides a useful sampling of users. Our complete study included 81 participants, aiming to investigate significant differences between the experimental groups and to train confusion detection models using multimodal data. For the release of raw data presented here, 28 out of 81 participants consented to the public release of their raw data under specific conditions for research purposes only. Notably, however, a sample size analysis [18,19] shows that a total sample size of 27 is recommended for two dependent experimental groups, hence, the raw data publication here is of use in a wide class of model creation and testing. It is also worth noting that the remaining data has been released as anonymised feature data in [9].

## 3 Data Description

**Fig. 1:**
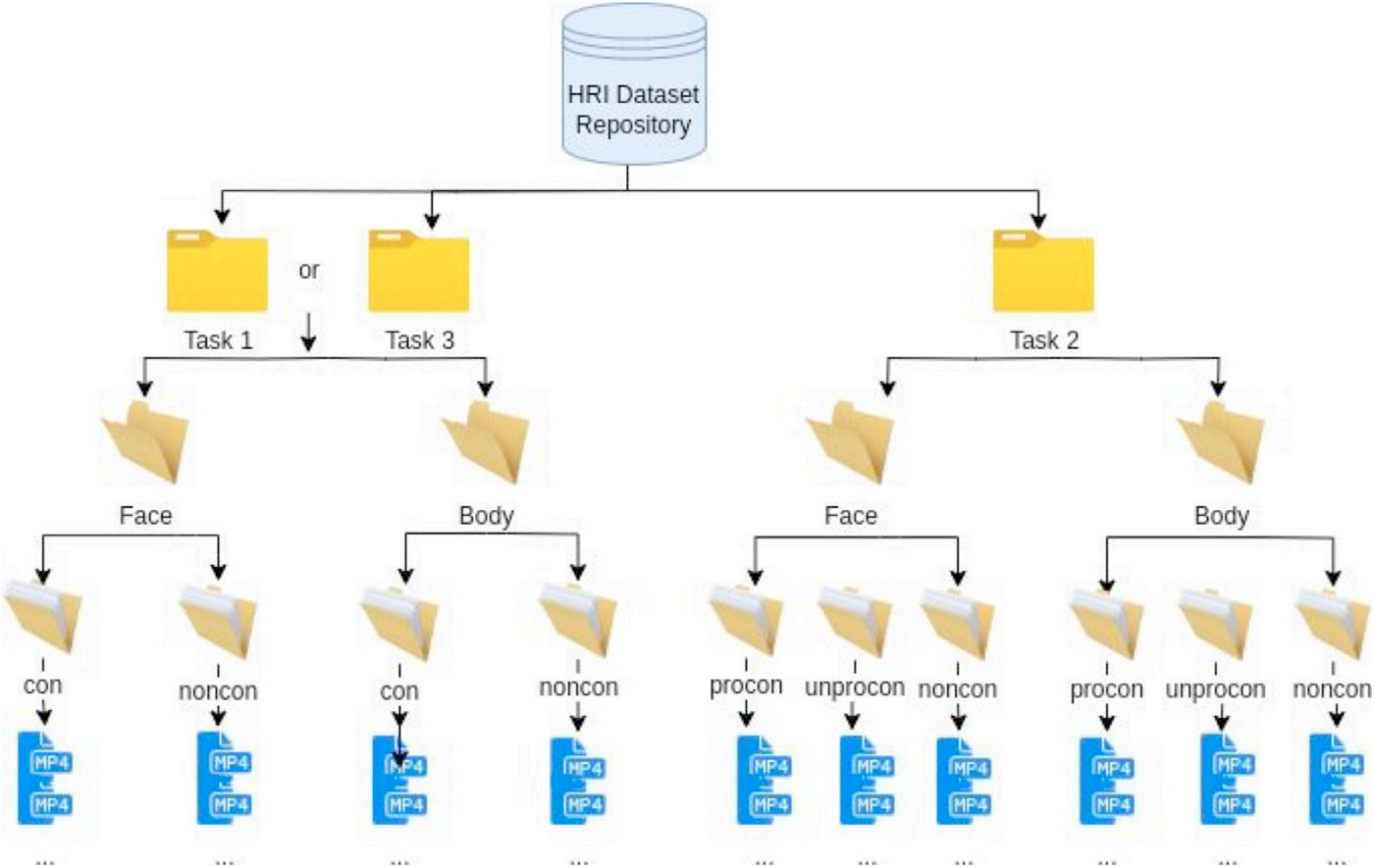
Folder structure of HRI dataset (con: confusion, noncon: non-confusion, procon: productive confusion, unprocon: unproductive confusion.).

This dataset contains three tasks, organised into three folders within the repository (see Fig. 1), in each task folder, facial videos (MP4) and body videos (MP4) are stored in two separate folders. Each facial or body language video corresponds to a single interactive conversation under one experimental condition. Within each “face” and “body” folder, the data are further categorised into subfolders based on the experiment conditions. Participants, who are from eight countries and work or study in Ireland, contribute these data. All participants are over 18 years of age, with the following age distribution: 1 participant aged 60+, 11 participants aged 18 - 24, 11 participants aged 25 - 44, and 5 participants aged 45 - 59. Table 1 provides the details of the number of videos with three experimental conditions for each task.

**Table 1:** The number of videos with different conditions.

Type	Task 1		Task 2			Task 3		Total
	con	noncon	procon	unprocon	noncon	con	noncon	
**body**	37	43	40	60	104	54	54	392
**face**	37	44	36	68	104	54	54	397

con: confusion, noncon: non-confusion, procon: productive confusion, unprocon: unproductive confusion.

### 3.1 Data request process

These raw data are not anonymous – containing human face, body and speech data – and are not shared publicly. Thus, in accordance with GDPR and to protect user data, access to the raw data can be requested by submitting an application form to the authors (application form: https://sites.google.com/view/hridatarequst/home).

The data request application includes a document outlining the copyright for User Confusion HRI dataset and an application form. Applicants must provide basic information (*e.g.*, name, email, job, etc.) and a brief description of the specific application for using the data. They must also agree that the data will be used solely for non-commercial, non-profit educational and research purposes.

We will evaluate each application and, if the criteria are met, will share a temporary download link with the applicant via email. The download link will be valid for five working days.

## 4 Experimental Design, Materials and Methods

This dataset was acquired by recording users’ multimodal behaviours using a controlled methodology, in which users interacted with a humanoid robot (Pepper) to perform the three designed tasks. In each task, language-based, semi-automated, one-to-one, interactions were designed to induce either confusion states or non-confusion using a WoZ (Wizard-of-Oz) controlled methodology [11]. This approach means that the Pepper robot was fully controlled by the researcher during experimental interactions. Task 1 represents an iterative and enhanced version of our initial pilot study [8], employing the same dialogue scripts. In this task, the Pepper robot asked three types of questions as part of task-oriented interactions: logical problems, math questions, and word problems. Each type of question was designed with two experimental conditions (*i.e.,* confusion and non-confusion). The key difference in this study is that participants evaluate their confusion levels after each dialogue. Additionally, all experiment devices and the experiment environment were improved.

Task 2 represents a more fine-grained approach to confusion invocation than in Task 1, focusing on a single type of question (word problems) [9], This task, however, includes three experimental conditions: productive confusion, unproductive confusion and non-confusion.

Task 3 involved a pick-and-place blocks game, in which the robot instructed users to pick up and place the four blocks into or out of three boxes. Confusion may be induced during the performance of these physical tasks, depending on the robot’s requests. The dialogue design for pick and place game is provided here.^1^

**Fig. 2:**
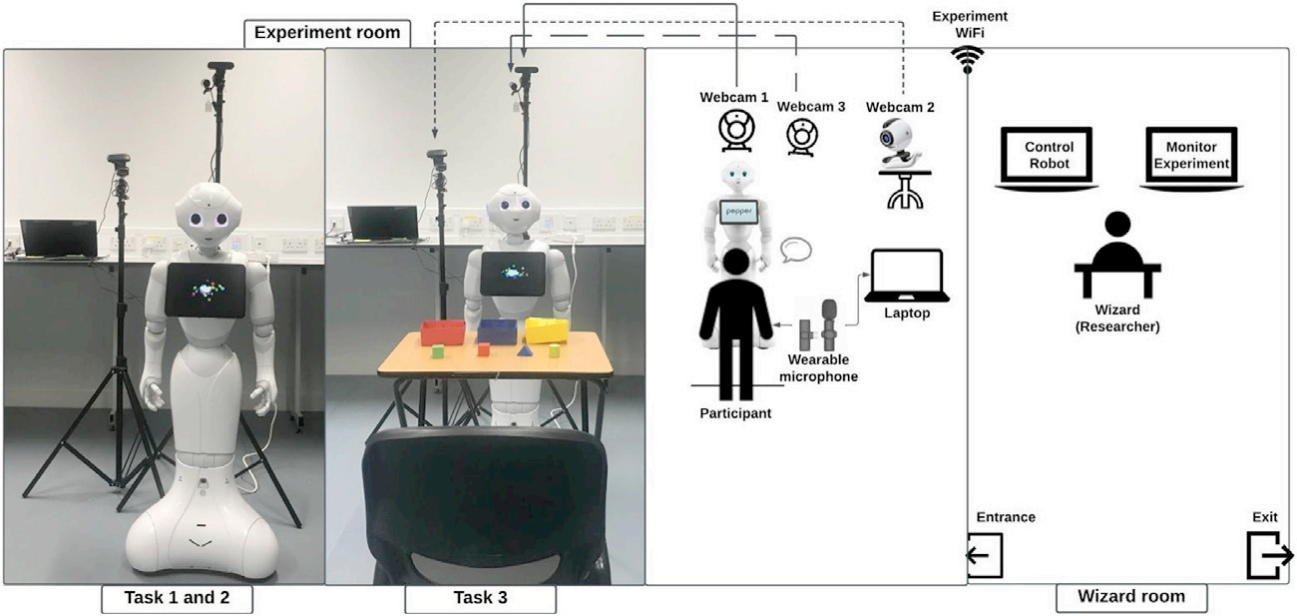
Experiment setup for Tasks 1, 2, and 3.

Turning to the experiment setup, we developed a robotics platform using the Naoqi robotic framework, an open programmable platform for robot control, as well as for implementing multi-interactive behaviours and animated speech in English. The robot’s body language corresponds to its speech is vivid, and we also designed the robot’s gestures to convey positive and negative responses [8]. Fig. 2 illustrates the experimental setup for the three tasks.

In Tasks 1 and 2, the participant stood in front of the Pepper robot for verbal interactions. In Task 3, as participants needed to perform pick-place tasks during conversations with the robot, which involved more than purely verbal interactions, we asked participants to sit at the front of the experiment table. This arrangement encouraged effective collaboration between the participant and the robot and allowed us to capture participants’ faces more clearly than if they had lowered their heads to complete a task. The experiment table was placed between the participant and the Pepper, with three boxes (red, blue and yellow) and four blocks arranged from left to right in front of the participant: one green cube, one red cube, a blue triangular prism, and a yellow cylinder.

The method of data collection is based on the use of two webcam and one microphone to record user behaviours from different perspectives: the first webcam was placed behind the robot, facing the participant’s face (face videos); the second webcam (right) was positioned to the right of the robot to record the participant’s body (body videos); the microphone was set for collecting user speech. An additional webcam was mounted on the same tripod as the first webcam to allow the researcher in the Wizard Room to monitor the experiment. All webcams and the microphone were connected to an experimental laptop in the experiment room.

**Fig. 3:**
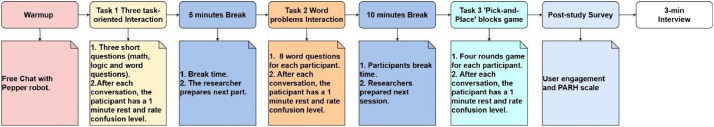
Experiment process.

Regarding the experiment process, each participant completed eight experiment sessions, which lasted around 60-min (see Fig. 3). There are two types of interaction: the first session involves a free conversation with the Pepper robot for 5-min without recording. This allows the participant to experience interaction with the robot and to adapt to the robot’s speech. During this interaction, the Pepper operates in full-automated mode. The second interaction consists of experiment conversations^2^ with recording, which includes the three tasks, and the Pepper controlled by the researchers. After each designed interaction, the participant has a 1-minute rest and rates their confusion levels for the interaction that just ended. After the three tasks were completed, the study concluded with a post-study survey in which the participants evaluate their overall engagement in the experiment, followed by a 3-minute interview. During this interview, the researcher collects direct user feedback regarding their interactions with the social robot.

## Limitations

None.

## Ethics Statement

The relevant informed consent was obtained from those subjects. The data release has approval from the Data Protection Office in Technological University Dublin, Ireland, 2024, DPIA (Data Protection Impact Assessment) ID: HRI-Human Robot Interaction-117; Ethical clearance following GDPR (General Data Protection Regulation) for this study was obtained from the Research Ethics Committee in Technological University Dublin, Ireland, 2021, Protocol No. REC-20–168.

## CRediT Author Statement

**Na Li:** Conceptualization, Investigation, Data Curation, Writing - Original Draft. **Jane Courtney:** Writing - Review & Editing, Supervision. **Robert Ross:** Writing - Review & Editing, Supervision.

## Data Availability

HRI-Confusion (Reference data)

## Footnotes

1 https://github.com/nalibjchn/SituatedHRITrackConfusion/blob/main/pickandplace-tasks_scripts.pdf.

2 https://github.com/nalibjchn/SituatedHRITrackConfusion/blob/main/DialogueScripts.pdf.

## References

[1] A. Ben Youssef, C. Clavel, S. Essid, M. Bilac, M. Chamoux, and A. Lim, UE-HRI: a new dataset for the study of user engagement in spontaneous human-robot interactions, (2017), pp. 464–472.

[2] Ben Youssef A., Varni G., Essid S., Clavel C. (2019). On-the-fly detection of user engagement decrease in spontaneous human–robot interaction using recurrent and deep neural networks. Int. J. Soc. Robot..

[3] O. Celiktutan, S. Profile, H. Gunes, and E. Skordos, Multimodal human-human-robot interactions (MHHRI) dataset for studying personality and engagement intelligent, (2017).

[4] D’Mello S., Graesser A. (2014). Confusion and its dynamics during device comprehension with breakdown scenarios. Acta Psychol.

[5] Day Alex, Karamouzas Ioannis (2023). A study in Zucker: insights on interactions between humans and small service robots. IEEe Robot. Autom. Lett..

[6] A. Gupta, R. Jaiswal, S. Adhikari, and V.N. Balasubramanian, Daisee: dataset for affective states in e-learning environments, ArXiv, abs/1609.01885 (2016).

[7] Li N., Kelleher J.D., Ross R. (2021). 25th Workshop on the Semantics and Pragmatics of Dialogue (SemDial 2021) University of Potsdam.

[8] Li N., Ross R. (2023). Proceedings of the 2023 ACM/IEEE International Conference on Human-Robot Interaction, HRI ’23.

[9] Li N., Ross R. (2023). Invoking and identifying task-oriented interlocutor confusion in human-robot interaction. Front. Robot. AI..

[10] Lodge J.M., Kennedy G., Lockyer L., Arguel A., Pachman M. (2018). Understanding difficulties and resulting confusion in learning: an integrative review. Front Educ.

[11] L. Riek, Wizard of oz studies in HRI: a systematic review and new reporting guidelines, in HRI 2012, (2012).

[12] Stiber M., Bohus D., Andrist S. (2024). Proceedings of the 26th International Conference on Multimodal Interaction, ICMI ’24.

[13] Wang X., Kwon T., Rad M., Pan B., Chakraborty I., Andrist S., Bohus D., Feniello A., Tekin B., Frujeri F.V., Joshi N., Pollefeys M. (2023). Proceedings of the IEEE/CVF International Conference on Computer Vision (ICCV).

[14] J. Sweller, Element interactivity and intrinsic, extraneous, and germane cognitive load, 22 (2010), pp. 1–6.

[15] J.L. Plass, R. Moreno, and R. Brünken, Cognitive load theory, (2010).

[16] Cumbal R., Lopes J., Engwall O. (2020). Proceedings of the 2020 International Conference on Multimodal Interaction, ICMI ’20.

[17] Anderson E.C., Carleton R.N., Diefenbach M., Han P.K.J. (2019). The relationship between uncertainty and affect. Front. Psychol..

[18] Faul F., Erdfelder E., Lang A.G., Buchner A. (2009). Statistical power analyses using G*Power 3.1: tests for correlation and regression analyses. Behav. Res. Methods.

[19] Faul F., Erdfelder E., Lang A.G., Buchner A. (2007). G*Power 3: a flexible statistical power analysis program for the social, behavioral, and biomedical sciences. Behav. Res. Methods.

[20] Zehfroosh Ashkan (2017). 2017 25th Mediterranean Conference on Control and Automation (MED).

